# Evolution during Three Ripening Stages of Évora Cheese

**DOI:** 10.3390/foods9091140

**Published:** 2020-08-19

**Authors:** Graça P. Carvalho, Rute Santos, Anabela Fino, Paulo Ferreira, Francisco M. Rodrigues, João Dias

**Affiliations:** 1 Polytechnic Institute of Portalegre, Agrarian School of Elvas, 7350-092 Elvas, Portugal; 19253@ipportalegre.pt (A.F.); pferreira@ipportalegre.pt (P.F.); fmondragao@ipportalegre.pt (F.M.R.); 2 VALORIZA–Research Centre for Endogenous Resource Valorization, 7300-555 Portalegre, Portugal; 3 MED-Mediterranean Institute for Agriculture, Environment and Development, Núcleo da Mitra, Apartado 94, 7006-554 Évora, Portugal; 4 CEFAGE-Center for Advanced Studies in Management and Economics, Universidade de Évora, Palácio do Vimioso (Gab. 224), Largo Marquês de Marialva, 8, 7000-809 Évora, Portugal; 5 Polytechnic Institute of Beja, Agrarian School of Beja, Rua Pedro Soares, 7800-295 Beja, Portugal; joao.dias@ipbeja.pt; 6 Geobiosciences, Geobiotechnologies and Geoengineering (GeoBioTec), Faculdade de Ciências e Tecnologias, Universidade Nova de Lisboa, 2829-516 Caparica, Portugal

**Keywords:** cheese, ripening, sensory analysis

## Abstract

The variability and heterogeneity found in Évora cheeses, Protected Designation of Origin (PDO), can affect consumers’ choices. Assessing the ripening conditions and their effect can be helpful. To study the effect of ripening duration in Évora cheese PDO, sensory and chemical analyses were performed in cheese samples subjected to 30, 60, and 120 days of ripening under controlled conditions (temperature 14 to 15 °C and humidity 65 to 70%). Sensory analysis was conducted with a homogenous panel previously familiarized with the product after a short training period, and chemical analyses including pH, moisture, NaCl content, a_w_, and salt-in-moisture were determined. Panelists were able to distinguish the differences in the organoleptic characteristics of the three cheese stages, and chemical determinations showed significant differences between stages. Interrater agreement was higher in the sensory evaluation of cheeses with a longer maturation period. As expected, cheeses in the 120 days ripening period presented lower pH, moisture, and water activity and had higher salt-in-moisture content. This stage received the highest scores in hardness and color of the crust, intensity, pungency of the aroma, intensity of taste and piquancy, and firmness and granular characteristics of texture. Overall acceptance of cheese samples was positive, regardless of the ripening stage, which probably reflects both the homogeneity of taster profiles and the previous knowledge of this particular product. The degree of ripeness influences the physical, chemical, and sensory characteristics but does not affect the acceptance of this product by the consumer.

## 1. Introduction

According to the FAO, the production of ewe milk cheese in Portugal was 11,434 tons in 2014 [1]. Évora cheese is a traditional raw ewe’s milk cheese in the Alentejo region in South Portugal [2]. It is a small, hard paste, ripened cheese usually sold from 6 to 12 months after manufacture. It can also be sold after a period of 1–1.5 months of ripening, as half-ripened cheese. Évora cheese has had a PDO designation since 1994, and its sensory and physicochemical attributes are essential to ensure its particular genuine properties [3]. It is traditionally sold in small grocery stores and street markets, and it is widely consumed in the region itself. According to Rivara, cited by Rodrigues et al. [2], in the past these cheeses were used as a daily payment to farm workers. The same authors state that the extensive sheep milk production system, manufacturing process, and ripening conditions are mainly responsible for the specificity of Évora cheese. It is exclusively coagulated with thistle (*Cynara cardunculus* L.) aqueous extract according to the specifications established for traditional and ancestral knowledge of production and processing. The coagulant enzymes extracted from *C. cardunculus* L. flowers, cardosins, are aspartic proteinases and have a more intense secondary proteolytic action on αs- and β-casein in cheese than other coagulants, with an impact on the biochemical and sensory properties of cheese [4]. In the manufacturing of Évora cheese PDO, salt is typically added to the curd, but it can also be spread at the top and bottom surfaces of the cheese.

Milk composition, differences in salting procedures, and the variability in storage conditions are sources of heterogeneity that affect cheese quality. These conditions determine the physical and flavor attributes of cheese, which influence the preference of consumers [2]. This heterogeneity is more visible when the same cheese product is marketed in different ripening stages. The ripening (maturation) period of cheese can range from two weeks to two or more years, and during this period the flavor and texture characteristics of the variety develop. Ripening usually involves changes to the microflora of the cheese, including death and lysis of starter cells, development of an adventitious nonstarter microflora, and, frequently, the growth of a secondary microflora [5]. The biochemical changes that take place during ripening include the metabolism of residual lactose, lactate, and citrate as well as lipolysis and proteolysis. Following these primary changes, there are secondary biochemical reactions that are critical for the development of volatile flavor compounds, including the metabolism of fatty acids and amino acids. Proteolysis is considered the most complex and important of the primary biochemical changes that occur in most cheeses during ripening [2]. The ripening period and conditions have been shown to influence physical, chemical, and rheological parameters as well as sensory attributes of ripened ewe cheeses [6,7].

Descriptive Analysis is the second major class of sensory test methods. This method quantifies the perceived intensities of the sensory characteristics of a product and is the most comprehensive and informative sensory evaluation tool. This method is used to characterize a wide variety of product changes and research questions in food product development. The information can be used to reveal insights into the ways in which sensory properties drive consumer acceptance, information, and instrumental measures using statistical techniques such as regression and correlations. There are different variations and improvements in the techniques of descriptive analysis [8].

This study focused on the influence of ripening time on the chemical properties and sensory features of Évora cheese in order to contribute to increase the knowledge about the quality of this cheese.

## 2. Materials and Methods

Cheeses were sampled at a local small cheese factory in the Évora district. All cheeses were ripened in the same chamber under equal temperature (14 to 15 °C) and humidity (65 to 70%) conditions. The manufacturing process was the same, with salt being spread on the top and bottom surfaces of cheese. Ten units of cheese were sampled at three different ripening periods (120, 60, and 30 days) from the same batch of production, corresponding to stages 1, 2, and 3, respectively, adding to a total of 30 cheese units under analysis.

In each cheese unit, a sample of 25 to 30 g was grated, and the NaCl content, moisture content, and water activity were analyzed. The NaCl content was determined based on the chloride content by potentiometric titration of the chloride ions with a solution of silver nitrate (0.1 mol/L) according to the Volhard method [9]. This method consists of titrating silver ions (Ag^+^) in excess with a standard thiocyanate solution and using the conversion factor of 58.4 to calculate the concentration of sodium chloride in g NaCl/100 g. The moisture content was determined by the gravimetric method, following NP 3544/1987 [10]. A 5 g mass of grated cheese sample (±0.01) was dried in an oven at 101 ± 1 °C for 4 h, and it was mixed with sand previously treated with hydrochloric acid. Subsequently, successive drying was carried out for 1 h until two consecutive weightings did not differ by more than 0.5 mg or there was an increase of mass.

The salt-in-moisture (%S/W) was calculated by dividing the NaCl-in-cheese content (%, *wt*/*wt*) by the moisture content (%, *wt*/*wt*). The water activity (a_w_) was determined using a Hygropalm water activity measuring device (Rotronic AG, Bassersdorf, Switzerland) in a temperature-controlled chamber at 20 °C. The pH value of the cheese mass was assessed by an electrode with a specific cap for hard samples, with a Mettler Toledo FiveEasy™ pH Electrode model LE438 DS (Mettler Toledo, Columbus, OH, USA). All measurements were performed in triplicate, and the average value of each determination was considered.

A panel of 12 tasters was selected. At present, there are no officially trained panelists for Évora cheese PDO. For this study, tasters were chosen among middle-aged (46 to 55 years old) women with higher education, born and residing in the Alentejo region, and familiar with this particular product; therefore, they were regular consumers of Évora cheese. A 3-h training session was performed to explain to tasters the terminology used to describe the different attributes of cheese according to [11] as well as the scale used to classify each attribute. The authors chose to reduce the variability of factors that influenced consumers’ attitudes towards the product, and to assess the panelists’ ability to distinguish between the three ripening periods under analysis. Tasters were asked to evaluate 18 attributes (6 regarding appearance, 3 regarding aroma, 5 regarding taste, and 4 regarding texture) [12], and they were asked to classify their global appreciation of the product and intent of purchase. Each attribute was classified on a scale from 0 to 5. A translation of the score evaluation sheet is presented as Supplementary File S1. Tasting sessions were performed in an appropriate room under controlled temperature (20 °C) with sensory booths on consecutive days. Tasters were asked to evaluate four cheese samples in each session. Each sample was presented in an individual plate coded with a randomly selected three-digit number. Samples consisted of three slices of cheese, approximately 2 to 3 mm thick, and were accompanied by a half cheese to provide means for appearance attributes evaluation. Tasters were also provided with mineral water, non-salted crackers, and an apple slice to cleanse their palates between tastings. The experimental design was conceived in a monadically way so that, in each session, tasters would be presented with one sample from each ripening stage (stages 1, 2, and 3) and one blind duplicate sample. Therefore, each taster evaluated a total of 9 different cheese units and 3 duplicates, for a total of 12 samples. On the other hand, the design allowed for each cheese unit to be evaluated by at least three different tasters.

### Statistical Analysis

Shapiro–Wilk tests were used to evaluate the normality of data distribution. As data were not normally distributed, Kruskal–Wallis tests for independent samples followed by multicomparison post-hoc tests (we employed the Dunn test considering Bonferroni correction of significance) were used to assess differences between stages, and associations between variables were estimated using Spearman’s correlation coefficients. Intra- and inter-rater reliabilities for sensory analysis were calculated using intra-class correlation coefficients (ICCs). According to the definitions established by McGraw & Wong [13], ICC estimates and their 95% confidence intervals were calculated using a two-way fixed effects, consistency, mean ratings model for both intra- and inter-rater reliability. Missing data were excluded from the analysis. All tests were performed using SPSS statistical package version 26 (SPSS Inc., Chicago, IL, USA), and statistical significance was set at *p* < 0.05.

## 3. Results

### 3.1. Differences between Ripening Stages in Chemical Analysis

Significant differences were found between ripening stages in the five chemical determinations. Cheese samples in stage 1 (120 days of ripening) showed a significantly lower pH value than those in stages 2 (60 days of ripening) and 3 (30 days of ripening). The values of moisture, water activity, NaCl content, and salt-in-moisture were significantly different between the three stages (Table 1).

### 3.2. Differences between Ripening Stages in Sensory Analysis

The results of the Kruskal–Wallis test regarding sensory analysis scores between the three studied ripening stages are shown in Table 2. The panelists gave high scores to the following: uniformity—color, hardness of the rind; odor/aroma—intense and lactic; taste—intense, salty, acid, piquant, and lactic; and texture—firm. These characteristics are typical of this cheese; however, the scores varied according to the cheese’s maturation time in same attributes.

### 3.3. Intra- and Inter-Rater Reliability.

There were significant differences between scores in the three stages regarding hardness and color of the crust, which increased with the ripening time, and between the shape of the profile of stage 3 and the remaining stages. Statistical differences in scores regarding odor/aroma, taste, and texture attributes were found, in most cases, between cheese samples in stage 1, with increased ripening time, and the other two stages. No significant differences were found in scores attributed to the three stages regarding global appreciation and intent of purchase.

**Figure 1:**
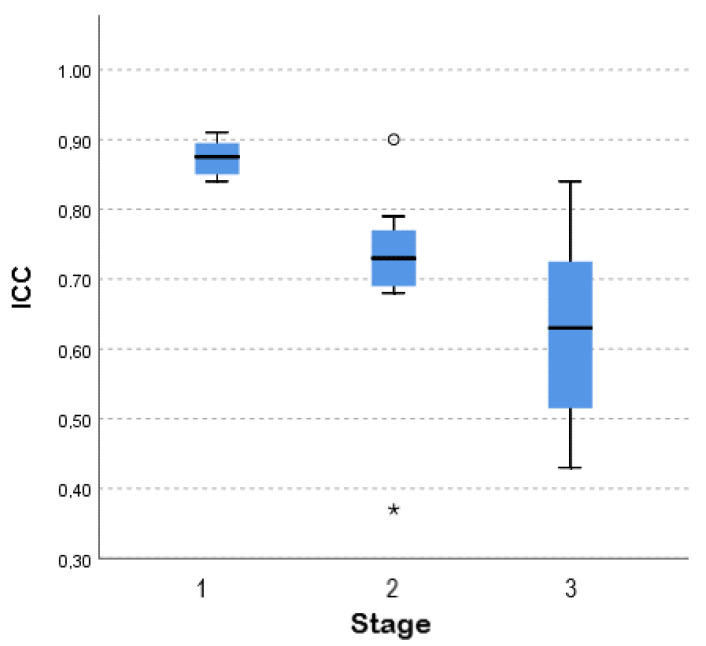
Median test for ICC according to stage. The dimension of the box refers to the interquartile range and vertical lines identify the set of observations which are non-outlier observations. ^ο^ and * represent, respectively, moderate and severe outliers.

All 12 raters unknowingly scored two or three duplicate samples from the same cheese. These scores were used to estimate ICC as a measure of intra-rater reliability. The large majority of ICC estimates indicated good intra-rater reliability (0.75 < ICC < 0.90), according to previously published recommendations [14]. Values are presented in Supplementary Material Table S2. ICC estimates were also obtained to measure inter-rater reliability, using the scores of 4 or 5 raters for 24 of the 30 cheese units in the sample (8 from each stage). With few exceptions, ICC estimates indicated moderate to good reliability in the consistency of rater’s scores concerning the same sample (0.50 < ICC < 0.9). Values are presented in Supplementary Material Table S3. To investigate whether inter-rater reliability differed between stages, a Kruskal–Wallis test for distributions and medians was performed, and it showed significant differences between the three stages (*p* = 0.001). The ICC median for stage 1 was significantly higher than for stage 2, which in turn was significantly higher than for stage 3 (Figure 1). It is important to highlight that, due to the outliers presented in stage 2, an apparent low difference between stages 2 and 3 was found.

### 3.4. Association between Variables

Spearman’s correlations between chemical determination values are shown in Table 3. Significant, positive associations were found between pH, moisture content, and water activity, and also between NaCl content and salt-in-moisture. On the other hand, significant, negative associations were found between NaCl content and moisture, and also between salt-in-moisture and pH, moisture, and water activity.

Spearman’s correlation coefficients between sensory analysis scores are presented in Supplementary Table S4. Significant correlations were found between the appearance attribute scores, such as hardness of the rind and color, and the majority of the remaining scores. Positive correlations were found between attributes associated with cheese maturation, and negative correlations were found with attributes associated with a shorter ripening period, such as lactic taste or buttery texture. Significant, positive correlations were found between the intensity of flavor (aroma and taste) and the global appreciation and intent of purchase.

Correlations between chemical determinations and sensory analysis scores are presented in Table 4. Regarding appearance attributes, hardness and color of the rind scores showed a positive association with NaCl content and salt-in-moisture as well as a negative association with pH and moisture. Despite the existence of some significant correlations, we need to highlight the fact that some of the correlations were not too strong, which implies some caution for the conclusions in this study.

## 4. Discussion

In the absence of a previously trained panel, and with detection of differences between the three ripening periods, we chose to screen a homogenous stage of tasters, previously familiar with the product, and provide a short training period to conduct an analytical sensory test using quantitative descriptive analysis. Quantitative Descriptive Analysis is considered the sensory tool of choice when establishing relationships with instrumental measurements. This method uses a trained set of individuals (generally 12) to identify and quantify specific sensory attributes, or all of the sensory attributes, of food [15]. Even though the panelists in this study did not undertake a full-length, comprehensive training period, they nevertheless showed good intra-rater reliability and moderate to good inter-rater reliability, according to Koo & Li [14]; thus, they showed surprisingly good consistency when unawaringly evaluating different samples of the same cheese and an adequate agreement among raters evaluating the same sample. These results probably show that previous knowledge and experience as consumers of the products under analysis have contributed to their consistent perception by panelists. Additionally, the fact that panelists were homogenous in age, gender, and socio-economic profile probably contributed to a similar vocabulary of attributes for similar perceptions of the same sample. However, and even though we consider these results showed that the sensory evaluations by these panelists were reliable, considering the aims of this particular study, they should nevertheless be considered with caution, as results obtained by a different subset of panelists may show appreciable differences from those reported.

When considering ICC values as a measure of interrater reliability, we obtained statistical differences regarding stages 1, 2, and 3. Panelists attributed more consistent scores to cheeses that had undergone a longer ripening period, and interrater consistency seems to decrease in cheeses in earlier ripening stages. Evidence suggests that the intensity of attributes, namely those related to flavor, is associated with higher agreement between raters [16]. This study was not designed as a consumer test, as the acceptance and preference of consumers were not the aims of the study, and the number of panelists was too low and inadequate for such a design [15].

The uniformity of the rind, smoothness, and presence of cracks in the rind showed no significant differences between stages. However, the color and hardness of the rind showed significant differences and a high, positive correlation with salt content and salt-in-moisture. Cheeses from Stages 1 and 2, with more than 60 days of ripening, showed differences in “shape of profile”, salty taste, and pungent odor compared with the cheeses from Stage 3 (30 days of ripening).

Ewe’s milk cheeses made from raw milk, such as those used in this study, are firmer and have a more characteristic odor, taste, and aftertaste than those manufactured with pasteurized milk [17]. The significant reduction of moisture content in the cheeses throughout the ripening period (Table 2) showed a strong, negative correlation with the firmness of texture (Table 4), in agreement with the results found by Tejada et al. [18]. The same occurred with saltiness and pungent odor. The apparent saltiness of cheese tended to increase with maturity, salt concentration, and decreasing pH [19]. There is an inverse relationship between salt levels and moisture, as was observed in this study, and consequently, there was a positive correlation with the S/W parameter. On the other hand, high salt concentrations are associated with increased levels of fat and protein due to water loss. These increases in fat and protein levels can also be induced by lipolysis and proteolysis phenomena as well as subsequent production of different kinds of products, such as volatile compounds produced during the metabolism of fatty acids and amino acids [6]. Several studies presented a relationship between the initial salt content in the matrix of cheese and changes in the composition and geometrical arrangement of casein, which in turn, influences the texture of cheese, namely regarding hardness, adherence, and viscosity [20,21,22,23,24]. Odor intensity showed a positive and significant correlation with S/W (Table 4), and cheeses in stage 1 were significantly different from the cheeses in stages 2 and 3 (Table 1) regarding odor intensity perception, which shows the influence of ripening on odor development. Our results confirm that more mature ewe’s milk cheeses are given higher scores for their characteristic and pungent attributes of flavor [17].

Salt is considered an important provider of cheese flavor and has been shown to increase the flavor intensity of ripened cheese while reducing bitterness [24]. According to El-Nimr et al. [24], texture and color are important criteria used to evaluate cheese quality; these two parameters are often the primary consideration of consumers when making purchasing decisions. However, in this study, the intent of purchase and the global appreciation results were not significantly different, showing the high-level satisfaction of tasters with any of the cheese stages, regardless of the stage of ripeness. Nevertheless, crust evaluations, namely hardness of the rind and color of the crust, showed significant differences between ripening stages, as both increased as the maturation time increased. The same was observed by Tejada et al. [18] for the color of cheeses after 90 days of ripening, compared with cheese after 60 days of ripening, even though significant differences (*p* < 0.05) were detected only in cheeses made with rennet.

There were significant differences between the three ripening stages for all the evaluated chemical parameters, showing a strong effect of ripening on Évora cheese PDO (Table 2). Pinheiro et al. [25] studied the proteolytic effect of aqueous extracts of *Cynara cardunculus* L, the vegetable coagulant typically required for Évora cheese, compared with a commercial animal rennet in sequential stages of ripening, and they established a degradation pattern of the casein fractions. Galán et al. [26] used powdered vegetable coagulant from *C. cardunculus* flowers, in comparison with calf rennet, and studied the changes in the chemical, biochemical, and sensory characteristics during the ripening process of ewe’s milk cheese as a means of reducing ripening time. This vegetable coagulant contains a mixture of acid or aspartic proteinases (endopeptidases), the same type of enzymes used for cheesemaking, such as chymosin, pepsin, and some other proteases of microbial origin [5]. The proportion of total soluble nitrogen (SN) has traditionally been regarded as a ‘ripening index’ for cheese as it correlates with the extent of proteolysis. The higher contents of tyrosine and tryptophan, which are soluble amino acids (Tyr *p* > 0.05, Trp *p* < 0.001), in the cheese samples made with vegetable rennet reflect the extent of proteolysis since, at least in the case of tyrosine, it was significantly related to SN (*p* < 0.001) in both types of rennet [27].

The concentration of NaCl in cheese (generally varying from 0.7 to 4%, an equivalent to 2–10% salt in the moisture phase) is sufficient to halt the growth of starter bacteria [28]. High salt levels tend to cause curdy textures, probably due to insufficient proteolysis; a pasty body, often accompanied by off-flavors, has been associated with low salt and high moisture levels. Previous research indicates that high humidity and pH values and a low salt level lead to taste and texture defects [29].

The results of the sensory analysis showed significant correlations between texture parameters and salt-in-water, which were positive for firmness and granular texture and negative for pasty and buttery texture (Table 4). According to Inhyu et al. [23], quoting Tomas & Pierce (1985), the NaCl content exerts several important effects on the textural aspects of cheese, initially enhancing protein hydration but, in high concentrations, is associated with a decrease in casein hydration and affects the cheese matrix to become firmer and stiffer, as in this study.

Concerning the pH results, the level was generally very low and decreased over the ripening period until 120 days, showing the effect of different factors that occur simultaneously. Sanjuan et al. [30] observed a significantly decreasing trend of pH in a Spanish artisanal ewe’s cheese type and an increase of the acid lactic level with ripening. Galán et al. [26] also observed a reduction in pH value until 60 days of ripening but this was followed by an increase until 180 days of ripening. According to Guinee [28], the level and time of salting have a major influence on pH changes in cheese.

The relative concentrations of different peptides and amino acids are variable and unique to particular cheese varieties. In addition, differences in the actions of these proteolytic agents cause differences in peptide profiles, as previously mentioned [5]. Peptides may have a direct impact on cheese flavor (some are bitter) or may provide a brothy background flavor to cheese [5]. The pH of cheese directly affects the texture of curd by influencing the solubility of the caseins; high-pH cheeses are softer than more acidic cheeses, if all other conditions are the same [31]. Salt, pH, and calcium content exert direct influences on the extent of hydration or paracasein aggregation, which, in turn, affects the capacity of hydration of the product’s matrix and its tendency to syneresis [28].

All samples showed a low water activity level with significant differences between them, although a higher value was observed at 60 days of ripening, coinciding with a higher salt content. However, the S/W increased throughout the ripening period in all cheese stages and is in agreement with the results of Wemmenhove et al. [32], showing the influence of moisture content. A decline in the average water activity was observed during ripening, concomitant with a reduction of the water-in-cheese content. It is generally considered that a water activity level under 0.92 is necessary to prevent bacterial growth; this is equivalent to a salt concentration of ~12%. In cheese, salt concentration varies from 0.7 to 7% [33].

## 5. Conclusions

Ripening conditions, particularly the maturation period, have a significant effect on both sensory scores and chemical parameters of Évora cheese PDO. The main impacted variables by ripening were, for the physical–chemical parameters, the moisture content and the salt-in-moisture and, for the sensory analysis, the textural attributes, characteristics of the rind, and the odor/aroma. The use of raw ewe’s milk and vegetable coagulant in the manufacturing of this cheese confer its characteristic profile, which is markedly influenced by the maturation period. Sensory analysis was performed by a homogenous set of consumers who were familiar with the product and were subject to a short training period. The agreement between panelists improved as the maturation period of cheese increased. Overall acceptance of cheese samples was positive, regardless of the ripening stage, which probably reflects both the homogeneity of taster profiles and the previous knowledge, and an emotional relationship with this particular product. Additional research should focus on consumer ‘preferences, performing affective tests, that can help manufacturers to identify consumers’ regarding the maturation of Évora cheese PDO.

## Figures and Tables

**Table 1:** Descriptive statistics (Median ± Interquantile Range) for chemical determinations, overall and by ripening stage, and differences between ripening stages.

Parameters	N	KW Test Value	Stage 1	Stage 2	Stage 3
pH	90	49.5 **	4.6 ± 0.3 ^a^	5.0 ± 0.1 ^b^	5.0 ± 0.1 ^b^
%Moisture	90	127.3 **	20.7 ± 1.0 ^a^	41.0 ± 2.5 ^b^	53.3 ± 1.0 ^c^
a_w_	90	75.1 **	0.8 ± 0.01 ^a^	0.9 ± 0.1 ^b^	0.9 ± 0.02 ^c^
%NaCl	90	117.7 **	2.3 ± 0.2 ^a^	2.6 ± 0.6 ^b^	1.6 ± 0.3 ^c^
%S/W	90	127.3 **	11.2 ± 0.5 ^a^	6.3 ± 1.1 ^b^	3.0 ± 0.5 ^c^

Kruskal–Wallis X^2^ (chi squared) tests were reported for each parameter (** *p* < 0.01) followed by Dunn’s multiple post-hoc tests among pairwise mean ranks. Different superscript letters represent significant differences (*p* < 0.05).

**Table 2:** Descriptive statistics (Median ± Interquantile Range), overall and by ripening stage, and differences between ripening stages in sensory analysis scores.

Items	N	KW Test Value	Stage 1	Stage 2	Stage 3
Appearance					
Uniformity of the rind	135	0.715	3.0 ± 2.0	3.0 ± 2.0	3.0 ± 2.0
Smoothness of the rind	135	0.143	2.0 ± 2.0	2.0 ± 2.0	2.0 ± 2.0
Presence of surface cracks	134	2.461	1.0 ± 2.0	1.0 ± 2.0	2.0 ± 2.0
Hardness of the rind	132	74.730	5.0 ± 1.0 ^a^	3.0 ± 1.0 ^b^	1.0 ± 1.0 ^c^
Color of the rind	131	75.595	3.0 ± 1.0 ^a^	2.0 ± 1.0 ^b^	1.0 ± 1.0 ^c^
Shape of profile	126	20.904	2.0 ± 2.0 ^a^	1.0 ± 1.3 ^a^	3.0 ± 1.5 ^b^
Odor/aroma					
Intense	135	33.182 **	4.0 ± 1.0 ^a^	3.0 ± 1.0 ^b^	2.0 ± 1.0 ^b^
Lactic	135	24.713 **	1.0 ± 2.0 ^a^	2.0 ± 2.0 ^b^	3.0 ± 2.0 ^c^
Pungent	135	9.459 **	2.0 ± 3.0 ^a^	2.0 ± 3.0 ^a,b^	1.0 ± 2.0 ^b^
Taste					
Intense	133	29.691 **	4.0 ± 1.0 ^a^	3.0 ± 2.0 ^b^	3.0 ± 2.0 ^b^
Salty	136	10.682 **	3.0 ± 2.0 ^a^	3.0 ± 2.0 ^a^	2.0 ± 1.0 ^b^
Acidic	136	0.357	2.0 ± 1.0	3.0 ± 1.0	3.0 ± 1.3
Piquant	136	29.762 **	3.0 ± 2.0 ^a^	2.0 ± 2.0 ^b^	1.0 ± 2.0 ^c^
Lactic	134	33.830 **	1.0 ± 2.0 ^a^	2.0 ± 2.0 ^b^	3.0 ± 2.0 ^c^
Texture					
Firm	135	98.641 **	5.0 ± 1.0 ^a^	3.0 ± 1.0 ^b^	1.0 ± 1.0 ^c^
Grainy	136	25.906 **	3.0 ± 2.0 ^a^	2.0 ± 2.0 ^b^	2.0 ± 2.0 ^b^
Pasty	136	57.578 **	0.0 ± 0.0 ^a^	0.0 ± 1.0 ^b^	2.0 ± 2.0 ^c^
Buttery	136	80.940 **	0.0 ± 0.0 ^a^	1.0 ± 2.5 ^b^	4.0 ± 2.0 ^c^
Overall					
Global appreciation	136	4.541	4.0 ± 1.5	3.0 ± 1.0	3.0 ± 2.0
Intent of purchase	136	4.696	4.0 ± 1.0	3.0 ± 1.0	3.0 ± 2.0

Kruskal–Wallis X^2^ (chi squared) tests were reported for each parameter (** *p* < 0.01) followed by Dunn’s multiple post-hoc tests among pairwise mean ranks. Different superscript letters represent significant differences (*p* < 0.05).

**Table 3:** Spearman’s correlations among chemical parameters.

	pH	%Moisture	a_w_	%NaCl	%S/W
**pH**	1.000	0.557 **	0.367 **	0.013	−0.489 **
%Moisture	0.557 **	1.000	0.399 **	−0.507 **	−0.934 **
a**_w_**	0.367 **	0.399 **	1.000	0.119	−0.486 **
%NaCl	0.013	−0.507 **	0.119	1.000	0.601 **
**%S/W**	−0.489 **	−0.934 **	−0.486 **	0.601 **	1.000

** means a significant correlation at the *p* < 0.01 level.

**Table 4:** Spearman’s correlations between sensory analysis scores and chemical parameters.

Sensory Analysis Attributes		pH	%Moisture	a_w_	%NaCl	%S/W
Appearance	Hardness of the crust	−0.399 **	−0.724 **	−0.188 *	0.504 **	0.712 **
	Color of the crust	−0.419 **	−0.720 **	−0.234 **	0.411 **	0.714 **
	Shape of profile	−0.120	0.195 *	−0.221 *	−0.362 **	−0.185 *
Odor/Aroma	Intense	−0.184 *	−0.459 **	−0.199 *	0.186 *	0.450 **
	Lactic	0.312 **	0.390 **	0.217 *	−0.284 **	−0.456 **
	Pungent	−0.042	−0.279 **	−0.203 *	0.263 **	0.314 **
Taste	Intense	−0.295 **	−0.383 **	−0.264 **	0.161	0.451 **
	Salty	−0.146	−0.202 *	0.063	0.199 *	0.173 *
	Piquant	−0.334 **	−0.456 **	−0.184 *	0.209 *	0.436 **
	Lactic	0.208 *	0.459 **	0.226 **	−0.359 **	−0.513 **
Texture	Firm	−0.476 **	−0.828 **	−0.367 **	0.437 **	0.821 **
	Grainy	−0.326 **	−0.364 **	−0.176 *	−0.034	0.315 **
	Pasty	0.361 **	0.622 **	0.176 *	−0.433 **	−0.621 **
	Buttery	0.468 **	0.769 **	0.339 **	−0.470 **	−0.769 **
Overall	Global appreciation	−0.001	−0.140	−0.091	0.104	0.170 *
	Intent of purchase	0.039	−0.143	−0.086	0.115	0.174 *

*—significant at *p* < 0.05; **—significant at *p* < 0.01.

## Supplementary Materials

The following are available online at https://www.mdpi.com/2304-8158/9/9/1140/s1, Table S1: Evaluation sheet (translated to English), Table S2: Intrarater reliability: Intra-class Correlation Coefficients (ICC), 95% confidence intervals and results of the F test, Table S3: Interrater reliability: Intra-class Correlation Coefficients (ICC), 95% confidence intervals and results of the F test. Table S4: Spearman’s correlation coefficients between sensory analysis scores.
