# Supplementary material for: Evolution during Three Ripening Stages of Évora Cheese
Source: Foods. 2020 Aug 19;9(9):1140. doi: 10.3390/foods9091140 (PMC7555954; doi:10.3390/foods9091140)
Supplement: Supplementary file 1 [file foods-09-01140-s001.zip › S1.pdf]

SENSORY ANALYSIS OF ÉVORA CHEESE
EVALUATION SHEET – S1

POLITÉCNICO DE PORTALEGRE
Escola Superior Agrária de Elvas

CFD4 CHEESE

Date:_____/______/__________
Taster code: _______________

CRUST

APPEARENCE

	0	1	2	3	4	5
Uniformity	☐ Not at all	☐	☐	☐	☐	☐ Very much
Smoothness	☐ Not at all	☐	☐	☐	☐	☐ Very much
Cracks	☐ None	☐	☐	☐	☐	☐ Many
Hardness	☐ Very soft	☐	☐	☐	☐	☐ Very hard
Color	☐ Light	☐	☐	☐	☐	☐ Dark
Shape	☐ 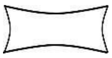	☐	☐	☐	☐	☐ 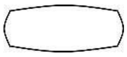

ODOUR/AROMA

	0	1	2	3	4	5
Intense	☐ Not at all	☐	☐	☐	☐	☐ Very
Lactic	☐ Not at all	☐	☐	☐	☐	☐ Very
Animal	☐ Not at all	☐	☐	☐	☐	☐ Very

TASTE

	0	1	2	3	4	5
Intense	☐ Not at all	☐	☐	☐	☐	☐ Very
Salty	☐ Not at all	☐	☐	☐	☐	☐ Very
Acidic	☐ Not at all	☐	☐	☐	☐	☐ Very
Piquant	☐ Not at all	☐	☐	☐	☐	☐ Very
Lactic	☐ Not at all	☐	☐	☐	☐	☐ Very

TEXTURE

	0	1	2	3	4	5
Firm	☐ Not at all	☐	☐	☐	☐	☐ Very
Grainy	☐ Not at all	☐	☐	☐	☐	☐ Very
Pasty	☐ Not at all	☐	☐	☐	☐	☐ Very
Buttery	☐ Not at all	☐	☐	☐	☐	☐ Very

	0	1	2	3	4	5
GLOBAL APPRECIATION	☐ I don’t like it	☐	☐	☐	☐	☐ I like it very much
INTENT OF PURCHASE	☐ I would never buy it	☐	☐	☐	☐	☐ I would prefer it to any other
