# Supplementary material for: Evolution during Three Ripening Stages of Évora Cheese
Source: Foods. 2020 Aug 19;9(9):1140. doi: 10.3390/foods9091140 (PMC7555954; doi:10.3390/foods9091140)
Supplement: Supplementary file 1 [file foods-09-01140-s001.zip › S2.docx]

**Table S2** – Intra-rater reliability: Intra-class Correlation Coefficients (ICC), 95% confident intervals and results of the F test

| Rater | ICC | Confident interval 95% | | F test | | | |
| --- | --- | --- | --- | --- | --- | --- | --- |
|  |  | Lower limit | Upper limit | Value | df1 | df2 | Sign. |
| 1 | 0,940 | 0,853 | 0,976 | 16,794 | 20 | 20 | 0,000 |
|  | 0,934 | 0,837 | 0,973 | 15,090 | 20 | 20 | 0,000 |
|  | 0,379 | -0,530 | 0,748 | 1,611 | 20 | 20 | 0,147 |
| 2 | 0,901 | 0,757 | 0,960 | 10,130 | 20 | 20 | 0,000 |
|  | 0,934 | 0,838 | 0,973 | 15,212 | 20 | 20 | 0,000 |
|  | 0,853 | 0,638 | 0,940 | 6,808 | 20 | 20 | 0,000 |
| 3 | 0,858 | 0,650 | 0,942 | 7,048 | 20 | 20 | 0,000 |
|  | 0,874 | 0,689 | 0,949 | 7,924 | 20 | 20 | 0,000 |
| 4 | 0,492 | -0,252 | 0,794 | 1,968 | 20 | 20 | 0,069 |
|  | 0,889 | 0,726 | 0,955 | 8,989 | 20 | 20 | 0,000 |
|  | 0,874 | 0,690 | 0,949 | 7,961 | 20 | 20 | 0,000 |
| 5 | 0,908 | 0,774 | 0,963 | 10,881 | 20 | 20 | 0,000 |
|  | 0,673 | 0,195 | 0,867 | 3,061 | 20 | 20 | 0,008 |
| 6 | 0,827 | 0,572 | 0,930 | 5,765 | 20 | 20 | 0,000 |
|  | 0,984 | 0,960 | 0,993 | 61,968 | 20 | 20 | 0,000 |
|  | 0,788 | 0,477 | 0,914 | 4,714 | 20 | 20 | 0,001 |
| 7 | 0,724 | 0,319 | 0,888 | 3,620 | 20 | 20 | 0,003 |
|  | 0,658 | 0,157 | 0,861 | 2,924 | 20 | 20 | 0,010 |
|  | 0,690 | 0,235 | 0,874 | 3,222 | 20 | 20 | 0,006 |
| 8 | 0,879 | 0,703 | 0,951 | 8,287 | 20 | 20 | 0,000 |
|  | 0,935 | 0,840 | 0,974 | 15,437 | 20 | 20 | 0,000 |
|  | 0,714 | 0,295 | 0,884 | 3,496 | 20 | 20 | 0,004 |
| 9 | 0,788 | 0,463 | 0,916 | 4,706 | 19 | 19 | 0,001 |
|  | 0,805 | 0,479 | 0,927 | 5,132 | 17 | 17 | 0,001 |
|  | 0,781 | 0,446 | 0,913 | 4,562 | 19 | 19 | 0,001 |
| 10 | 0,924 | 0,813 | 0,969 | 13,153 | 20 | 20 | 0,000 |
|  | 0,962 | 0,906 | 0,984 | 26,165 | 20 | 20 | 0,000 |
|  | 0,945 | 0,865 | 0,978 | 18,203 | 20 | 20 | 0,000 |
| 11 | 0,819 | 0,555 | 0,927 | 11,620 | 20 | 20 | 0,000 |
|  | 0,817 | 0,543 | 0,927 | 9,076 | 20 | 20 | 0,000 |
| 12 | 0,870 | 0,680 | 0,947 | 7,713 | 20 | 20 | 0,000 |
|  | 0,878 | 0,700 | 0,951 | 8,222 | 20 | 20 | 0,000 |
