# Supplementary material for: Evolution during Three Ripening Stages of Évora Cheese
Source: Foods. 2020 Aug 19;9(9):1140. doi: 10.3390/foods9091140 (PMC7555954; doi:10.3390/foods9091140)
Supplement: Supplementary file 1 [file foods-09-01140-s001.zip › S3.docx]

**Table S3** – Inter-rater reliability: Intra-class Correlation Coefficients (ICC), 95% confident intervals and results of the F test

| Group | Sample | ICC | Confident interval 95% | | F test | | | |
| --- | --- | --- | --- | --- | --- | --- | --- | --- |
|  |  |  | Lower limit | Upper limit | Value | df1 | df2 | Sign. |
| A | 1 | 0,909 | 0,826 | 0,959 | 10,964 | 19 | 76 | 0,000 |
|  | 2 | 0,882 | 0,777 | 0,946 | 8,455 | 20 | 80 | 0,000 |
|  | 3 | 0,847 | 0,718 | 0,930 | 6,554 | 20 | 100 | 0,000 |
|  | 4 | 0,842 | 0,702 | 0,928 | 6,318 | 20 | 80 | 0,000 |
|  | 5 | Insufficient data | | | | | | |
|  | 6 | 0,849 | 0,721 | 0,930 | 6,625 | 20 | 100 | 0,000 |
|  | 7 | Insufficient data | | | | | | |
|  | 8 | 0,866 | 0,739 | 0,940 | 7,443 | 19 | 57 | 0,000 |
|  | 9 | 0,896 | 0,795 | 0,954 | 9,621 | 19 | 57 | 0,000 |
|  | 10 | 0,894 | 0,790 | 0,953 | 9,426 | 19 | 57 | 0,000 |
| B | 1 | 0,754 | 0,515 | 0,892 | 4,072 | 19 | 57 | 0,000 |
|  | 2 | 0,899 | 0,800 | 0,956 | 9,899 | 19 | 57 | 0,000 |
|  | 3 | 0,790 | 0,586 | 0,908 | 4,768 | 19 | 57 | 0,000 |
|  | 4 | 0,746 | 0,499 | 0,889 | 3,941 | 19 | 57 | 0,000 |
|  | 5 | 0,706 | 0,446 | 0,866 | 3,403 | 20 | 80 | 0,000 |
|  | 6 | 0,681 | 0,402 | 0,856 | 3,137 | 19 | 95 | 0,000 |
|  | 7 | Insufficient data | | | | | | |
|  | 8 | 0,371 | -0,222 | 0,727 | 1,591 | 18 | 72 | 0,086 |
|  | 9 | Insufficient data | | | | | | |
|  | 10 | 0,703 | 0,440 | 0,864 | 3,365 | 20 | 80 | 0,000 |
| C | 1 | 0,711 | 0,395 | 0,883 | 3,457 | 16 | 48 | 0,000 |
|  | 2 | 0,431 | -0,124 | 0,750 | 1,758 | 19 | 57 | 0,052 |
|  | 3 | 0,843 | 0,706 | 0,929 | 6,386 | 19 | 95 | 0,000 |
|  | 4 | 0,586 | 0,219 | 0,811 | 2,413 | 20 | 80 | 0,003 |
|  | 5 | 0,579 | 0,207 | 0,808 | 2,376 | 20 | 80 | 0,003 |
|  | 6 | Insufficient data | | | | | | |
|  | 7 | 0,453 | -0,046 | 0,756 | 1,829 | 19 | 76 | 0,034 |
|  | 8 | Insufficient data | | | | | | |
|  | 9 | 0,739 | 0,485 | 0,885 | 3,836 | 19 | 57 | 0,000 |
|  | 10 | 0,671 | 0,351 | 0,856 | 3,043 | 19 | 57 | 0,001 |
