# Supplementary material for: Evolution during Three Ripening Stages of Évora Cheese
Source: Foods. 2020 Aug 19;9(9):1140. doi: 10.3390/foods9091140 (PMC7555954; doi:10.3390/foods9091140)
Supplement: Supplementary file 1 [file foods-09-01140-s001.zip › S4.docx]

**Table S4** – Spearman’s correlation coefficients between sensory analysis scores

|  | Uniform | Smooth | Cracks | Hard | Color | Shape | O-Inten | O-Lact | O-Animal | Inten | Salty | Acid | Piquant | Lact | Firm | Grain | Pasty | Butter | Gl.App. | Purch |
| --- | --- | --- | --- | --- | --- | --- | --- | --- | --- | --- | --- | --- | --- | --- | --- | --- | --- | --- | --- | --- |
| Uniform | 1.000 | 0.516^**^ | -0.185^*^ | -0.053 | -0.106 | 0.010 | -0.050 | -0.025 | -0.183^*^ | -0.044 | 0.077 | 0.007 | 0.032 | -0.036 | -0.018 | -0.010 | -0.057 | 0.066 | 0.083 | 0.063 |
| Smooth | 0.516^**^ | 1.000 | -0.019 | -0.140 | -0.037 | 0.150 | -0.136 | -0.035 | -0.402^**^ | -0.013 | 0.088 | -0.137 | -0.093 | 0.072 | 0.010 | -0.015 | -0.315^**^ | -0.095 | 0.149 | 0.119 |
| Cracks | -0.185^*^ | -0.019 | 1.000 | -0.144 | 0.017 | -0.072 | -0.212^*^ | -0.094 | 0.082 | -0.026 | -0.160 | 0.018 | -0.006 | -0.098 | -0.075 | -0.080 | 0.041 | 0.036 | -0.025 | -0.013 |
| Hard | -0.053 | -0.140 | -0.144 | 1.000 | 0.628^**^ | -0.250^**^ | 0.552^**^ | -0.300^**^ | 0.343^**^ | 0.378^**^ | 0.264^**^ | -0.015 | 0.416^**^ | -0.406^**^ | 0.683^**^ | 0.289^**^ | -0.471^**^ | -0.574^**^ | 0.308^**^ | 0.282^**^ |
| Color | -0.106 | -0.037 | 0.017 | 0.628^**^ | 1.000 | -0.143 | 0.374^**^ | -0.393^**^ | 0.398^**^ | 0.413^**^ | 0.114 | -0.115 | 0.321^**^ | -0.428^**^ | 0.712^**^ | 0.169 | -0.573^**^ | -0.629^**^ | 0.174^*^ | 0.191^*^ |
| Shape | 0.010 | 0.150 | -0.072 | -0.250^**^ | -0.143 | 1.000 | -0.081 | 0.235^**^ | -0.154 | 0.019 | 0.051 | -0.078 | -0.013 | 0.282^**^ | -0.176^*^ | 0.147 | 0.134 | 0.252^**^ | 0.116 | 0.058 |
| O-Inten | -0.050 | -0.136 | -0.212^*^ | 0.552^**^ | 0.374^**^ | -0.081 | 1.000 | -0.085 | 0.405^**^ | 0.426^**^ | 0.218^*^ | 0.108 | 0.439^**^ | -0.113 | 0.399^**^ | 0.329^**^ | -0.197^*^ | -0.319^**^ | 0.283^**^ | 0.329^**^ |
| O-Lact | -0.025 | -0.035 | -0.094 | -0.300^**^ | -0.393^**^ | 0.235^**^ | -0.085 | 1.000 | -0.039 | -0.215^*^ | -0.072 | 0.031 | -0.273^**^ | 0.677^**^ | -0.409^**^ | -0.015 | 0.305^**^ | 0.493^**^ | -0.052 | -0.038 |
| O-Animal | -0.183^*^ | -0.402^**^ | 0.082 | 0.343^**^ | 0.398^**^ | -0.154 | 0.405^**^ | -0.039 | 1.000 | 0.199^*^ | -0.031 | 0.036 | 0.229^**^ | -0.169 | 0.269^**^ | 0.009 | 0.029 | -0.209^*^ | 0.095 | 0.123 |
| Inten | -0.044 | -0.013 | -0.026 | 0.378^**^ | 0.413^**^ | 0.019 | 0.426^**^ | -0.215^*^ | 0.199^*^ | 1.000 | 0.295^**^ | 0.035 | 0.417^**^ | -0.321^**^ | 0.557^**^ | 0.320^**^ | -0.355^**^ | -0.353^**^ | 0.224^**^ | 0.208^*^ |
| Salty | 0.077 | 0.088 | -0.160 | 0.264^**^ | 0.114 | 0.051 | 0.218^*^ | -0.072 | -0.031 | 0.295^**^ | 1.000 | 0.194^*^ | 0.447^**^ | -0.106 | 0.224^**^ | 0.383^**^ | -0.076 | -0.102 | 0.001 | -0.026 |
| Acid | 0.007 | -0.137 | 0.018 | -0.015 | -0.115 | -0.078 | 0.108 | 0.031 | 0.036 | 0.035 | 0.194^*^ | 1.000 | 0.246^**^ | 0.134 | -0.141 | 0.110 | 0.111 | -0.016 | -0.263^**^ | -0.226^**^ |
| Piquant | 0.032 | -0.093 | -0.006 | 0.416^**^ | 0.321^**^ | -0.013 | 0.439^**^ | -0.273^**^ | 0.229^**^ | 0.417^**^ | 0.447^**^ | 0.246^**^ | 1.000 | -0.316^**^ | 0.463^**^ | 0.375^**^ | -0.075 | -0.353^**^ | 0.154 | 0.122 |
| Lact | -0.036 | 0.072 | -0.098 | -0.406^**^ | -0.428^**^ | 0.282^**^ | -0.113 | 0.677^**^ | -0.169 | -0.321^**^ | -0.106 | 0.134 | -0.316^**^ | 1.000 | -0.502^**^ | 0.021 | 0.325^**^ | 0.423^**^ | -0.109 | -0.077 |
| Firm | -0.018 | 0.010 | -0.075 | 0.683^**^ | 0.712^**^ | -0.176^*^ | 0.399^**^ | -0.409^**^ | 0.269^**^ | 0.557^**^ | 0.224^**^ | -0.141 | 0.463^**^ | -0.502^**^ | 1.000 | 0.368^**^ | -0.587^**^ | -0.739^**^ | 0.207^*^ | 0.192^*^ |
| Grain | -0.010 | -0.015 | -0.080 | 0.289^**^ | 0.169 | 0.147 | 0.329^**^ | -0.015 | 0.009 | 0.320^**^ | 0.383^**^ | 0.110 | 0.375^**^ | 0.021 | 0.368^**^ | 1.000 | -0.147 | -0.227^**^ | -0.085 | -0.095 |
| Pasty | -0.057 | -0.315^**^ | 0.041 | -0.471^**^ | -0.573^**^ | 0.134 | -0.197^*^ | 0.305^**^ | 0.029 | -0.355^**^ | -0.076 | 0.111 | -0.075 | 0.325^**^ | -0.587^**^ | -0.147 | 1.000 | 0.640^**^ | -0.216^*^ | -0.187^*^ |
| Butter | 0.066 | -0.095 | 0.036 | -0.574^**^ | -0.629^**^ | 0.252^**^ | -0.319^**^ | 0.493^**^ | -0.209^*^ | -0.353^**^ | -0.102 | -0.016 | -0.353^**^ | 0.423^**^ | -0.739^**^ | -0.227^**^ | 0.640^**^ | 1.000 | -0.091 | -0.083 |
| Gl.App. | 0.083 | 0.149 | -0.025 | 0.308^**^ | 0.174^*^ | 0.116 | 0.283^**^ | -0.052 | 0.095 | 0.224^**^ | 0.001 | -0.263^**^ | 0.154 | -0.109 | 0.207^*^ | -0.085 | -0.216^*^ | -0.091 | 1.000 | 0.934^**^ |
| Purch | 0.063 | 0.119 | -0.013 | 0.282^**^ | 0.191^*^ | 0.058 | 0.329^**^ | -0.038 | 0.123 | 0.208^*^ | -0.026 | -0.226^**^ | 0.122 | -0.077 | 0.192^*^ | -0.095 | -0.187^*^ | -0.083 | 0.934^**^ | 1.000 |

* - significant at p<0.05; ** - significant at p<0.01
